# Bioactive nanocomposite PLDL/nano-hydroxyapatite electrospun membranes for bone tissue engineering

**DOI:** 10.1007/s10856-014-5149-9

**Published:** 2014-01-24

**Authors:** Izabella Rajzer, Elżbieta Menaszek, Ryszard Kwiatkowski, Wojciech Chrzanowski

**Affiliations:** 1 Division of Materials Engineering, Department of Mechanical Engineering Fundamentals, ATH University of Bielsko-Biala, Willowa 2 Street, 43-309 Bielsko-Biała, Poland; 2 Department of Cytobiology, Collegium Medicum, UJ Jagiellonian University, Medyczna 9 Street, 30-068 Kraków, Poland; 3 Institute of Textile Engineering and Polymer Materials, Faculty of Materials and Environmental Sciences, ATH University of Bielsko-Biala, Willowa 2 Street, 43-309 Bielsko-Biała, Poland; 4 Faculty of Pharmacy, The University of Sydney, Sydney, NSW 2006 Australia

## Abstract

New nanocomposite membranes with high bioactivity were fabricated using the electrospinning. These nanocomposites combine a degradable polymer poly(l/dl)-lactide and bone cell signaling carbonate nano-hydroxyapatite (n-HAp). Chemical and physical characterization of the membranes using scanning electron microscopy, Fourier transform infrared spectroscopy and the wide angle X-ray diffraction evidenced that nanoparticles were successfully incorporated into the fibers and membrane structure. The incorporation of the n-HAp into the structure increased significantly the mineralization of the membrane in vitro. It has been demonstrated that after a 3-day incubation of composite membrane in the Simulated Body Fluid a continuous compact apatite layer was formed. In vitro experiments demonstrated that the incorporation of n-HAp significantly improved cell attachment, upregulated cells proliferation and stimulated cell differentiation quantified using Alkaline Phosphatase and OsteoImage tests. In conclusion, the results demonstrated that the addition of n-HAp provided chemical cues that were a key factor that regulated osteoblastic differentiation.

## Introduction

The effective regeneration of fully functional body tissues including bone structures is one of the major challenges in regenerative medicine. Current approaches include surgical reconstruction using autografts or allografts. These methods have some limitations that are associated with the availability of autografts, the risk of immunogenicity and infection [1]. To address these issues tissues can be engineered ex vivo, which allows ‘manufacturing’ a tissue substitute that match specifically the implantation site. Therefore tissue engineering approaches hold great promise for regenerative medicine. Main requirements for materials used as scaffolds for bone tissue engineering are: (i) biocompatibility, (ii) biodegradability with a controllable degradation time, (iii) suitable surface chemistry to regulate cell attachment, proliferation and differentiation, (iv) adequate mechanical properties that match those of tissue at the site of implantation and (v) bioactivity attributed to the formation of a biological carbonated apatite layer on the surface of the scaffold, which leads to better osseointegration and the enhanced formation of new bone tissue within a short period [2, 3]. Natural extracellular matrix (ECM) provides physical environment for cells to attach, grow, migrate, respond to signals and also gives the tissue its structural and therefore mechanical properties, such as rigidity and elasticity that is associated with the tissue functions [4]. Ideally the scaffold should mimic the structure of the fibrous component of the ECM. Many extracellular proteins have a fibrous structure with diameters on the nanometer or micrometer scales, surrounded and infiltrated by nano-sized crystals of apatite [5]. Nano-structured scaffold can improve the cell–matrix interaction by adsorption of cell adhesion-mediating molecules from biological fluids [6]. One of the techniques to manufacture nanofibrous scaffolds for tissue regeneration is electrospinning. This technique enables the fabrication of scaffolds with different topographies and porosities (at nano to microscale) inspired by ECM that are capable of controlling cellular responses [7]. Electrospun scaffold have high surface area and interconnected pore network, providing a facile transport of metabolic nutrients and waste through the nanometer-sized pores, whereas the efficient cell implantation and blood vessel invasion can be expected through the micrometer-sized pores [8]. Sufficient osseointegration between scaffold and bone tissue is another important factor that should be considered for orthopedic scaffolds [9]. It is well established that calcium and phosphates containing materials, i.e. hydroxyapatite (HAp), induce osteoblastic cell differentiation, thus osseointegration [10–13]. Therefore, the incorporation of the mineral phase into scaffolds can be utilized to modulate cellular responses and in consequence regenerate the tissue. The mineral phase, typically in the form of regular and irregular particles of different sizes, can be blended into polymer structures using electrospinning. This method result in physical blend of both phases and has fibrous structure with well controlled fibers diameter and interconnectivity, which makes electrospinning an attractive method for bone tissue engineering [14]. Several studies have investigated the effect of inorganic phase incorporated into biocompatible polymers on their morphology, mechanical properties and degradation behavior [15]. Addition of HAp particles into the nanofibrous scaffold during electrospinning resulted in enhancement in thermal and mechanical properties [16] as well as in improvement of scaffold bioactivity [17, 18]. The composite scaffolds exhibited better cell adhesion and growth than scaffold without bioactive nanoparticles [19] and also higher cell viability and ALP activity [20].

Poly(lactic acid) (PLA) has been frequently used in many orthopaedic applications. It can be easily processed into shapes such as screws, pins and plates for tissue fixation, sutures and surgical staples for wound closure and fabricated into scaffolds or devices for controlled delivery of biomolecules [21]. The success of PLA in biomedical applications is strictly connected with its tuneable degradation, which occurs by hydrolysis and sufficient mechanical properties [22, 23]. Co-polymers including poly(l/dl lactide) are clinically used materials for fracture fixation. The ratio of l and dl isomeric forms can vary and subsequently impact of material characteristic due to different level of crystallinity. For example greater level of crystllinity is observed for l/dl ratio is higher. Thus the use of amorphous poly(l/dl)-lactide 70:30 has a significant benefit because it prevents from the generation of undesired degradation products in the form of highly crystalline debris. It is also expected that by using this co-polymer the tissue reaction to the material will be milder. Implants produced from poly(l/dl-lactide) 70:30 are in clinical use for fracture fixation in regions of limited mechanical load and are well suited for the fabrication of scaffold for the regeneration of the bone tissue. Also the degradation time is suitable for such application as well as it characterises with good formability into the fibers. Hence, these characteristics make PLDL a good candidate for scaffold and it was utilized in this study [24, 25]. Hydroxyapatite is calcium phosphate mineral naturally occurring in human tissues, which is commonly used as a bone graft. One of the current approaches to improve biological properties of synthetic HAp is to adjust more closely its chemical composition and morphology to that of cancellous bone through incorporation of carbonate ions into the HAp structure (cHAp) [26]. Carbonate ions improve the solubility of HAp, thus its release on Ca and P ions that encourage bone regeneration. Hydroxyapatite is also often used as filler in polymer based composites to enhance their integration in bodily environment, buffer (when poorly crystalline HAp is used) degradation products and modify mechanical properties. Therefore, the incorporation of carbonate HAp particles into electrospun membranes could regulate osteoinductivity of the membranes, thus stimulate de nove bone tissue formation. It is well established that proteins adsorb very well to HAp, at the same time proteins are dominating signaling cascade that regulate cellular responses. Hence, it is highly advantageous to use HAp as filler for fibrous membranes that are used for tissue regeneration. The objective of this study was to fabricate an PLDL/n-HAp composite membrane using the electrospinning process. We investigate the effect of carbonated n-HAp addition on membranes properties and in vitro mineralization in Simulated Body Fluid. We also evaluated the attachment, proliferation and osteogenic differentiation of human primary NHOst cells cultured on electrospun composite nanofibrous membranes.

## Materials and methods

### Materials

Carbonate nano-hydroxyapatite was synthesized by a wet precipitation method as previously described [26]. An average size of the n-HAp particles was 23 nm and the specific surface area of the n-HAp powder was 79.9 m^2^/g. Co-polymer of l-lactide and dl-lactide (PLDL) was purchased from PURAC (The Netherlands). Acetone (POCH, Poland) was used as a solvent.

The electrospinning solutions were prepared by dissolving 1 g of PLDL copolymer in 50 ml of acetone (POCH, Poland) at room temperature under magnetic stirring. For the composite scaffold the PLDL–acetone solution was mixed with 20 wt% of n-HAp. Stable dispersion of n-HAp colloidal suspension was achieved by sonication of the solution.

#### Fabrication of the membrane by electrospinning

To fabricate the membranes electrospinning was used. Polymer solutions were loaded into a plastic syringe (20 mL) and injected through a stainless steel needle (diameter 0.7 mm) at injection rate of 1.5 mL/h. The needle was connected to a high voltage supply (30 kV). Rotating metal drum was place at a distance of 20 cm from the needle tip. Membranes were collected on silica coated paper attached to drum collector. Two types of nonwoven membranes were formed: PLDL without ceramic additives (PLDL) and PLDL modified with n-HAp (PLDL/n-HAp).

#### Membrane characterization

The morphology of fibrous membranes was characterized by scanning electron microscopy (SEM, Jeol, JSM 5500). Before SEM observation, all of the samples were cut from the electrospun membrane (0.5 × 0.5 cm) and then sputtered coated with gold (Jeol JFC 1200 sputter). Five SEM images were used for each fibrous sample to measure the average fiber diameter. From each image, at least 50 different fibers were selected.

Pore size distribution was measured using the capillary flow porometer (PMI, USA). For each sample pore size measurement was repeated three times. Isopropyl alcohol was used as a fluid medium.

Mechanical properties of the electrospun membranes were investigated using mechanical testing machine (Zwick-Roell Z 2.5) at 10 mm/min crosshead tension speed. The electrospun samples were carefully cut into a rectangular shape of about 20 mm width and 100 mm length. All membranes were weighed and their dimensions were measured with micrometer prior to testing. Five specimens were considered for each electrospun matrix. Tensile strength (UTS) was determined from the stress–strain curves.

The wide angle X-ray diffraction measurements (WAXD) were carried out on a Seifert URD6 diffractometer, equipped with ISO-DEBYEFLEX 3003 high voltage generator and a graphite monochromator. A cooper target sealed X-ray tube operated at U = 40 kV and I = 30 mA was used as the radiation source (λ = 1.542 Å). The step scanning measurement mode was employed over a 2θ scattering angle ranging from 5^o^ to 60^o^ and from 22.5^o^ to 37.5^o^, with a step-size of 0.05^o^.

Fourier transform infrared FTIR spectra were recorded using fotoacustic reflectance (MTEC Photoacoustics 300 THERMO NICOLET) at the range of 400–4,000 cm^−1^ using at least 64 scans and 4 cm^−1^ resolution.

#### Mineralization of scaffolds with simulated body fluid

1.5 × concentrated SBF was selected to examine mineralization process of PLDL/n-HAp and the PLDL membranes [27]. Samples (2.0 × 2.0 cm) were immersed in 15 mL of SBF and incubated at 37 °C for 14 days. The solution was renewed every two days to ensure sufficient ion concentration. After 1, 3, 7 and 14 days of incubation, the samples were rinsed with distilled water and then dried at room temperature. After each time point the surface morphology and membranes properties were examined by SEM, FTIR and WAXD methods.

Prior to cell culture studies the nanofibrous membranes were sterilized with 70 % ethanol for 10 min followed by exposure to ultraviolet (UV) light for half an hour each side.

### Cell study

#### Cell culture

Normal human osteoblasts (NHOst, Lonza, USA) were cultured in OGM culture medium, supplemented with 10 % FBS, ascorbic acid and 5 % solution of gentamicin and amphotericin-B (OGM BulletKit, Lonza, USA) in an atmosphere of 5 % CO_2_ at 37 °C. The tests were conducted on cells from passages 5 to 6. The cell suspension was obtained by addition of 5 % trypsin with EDTA (Lonza, USA). After flushing and centrifugation, the cells were concentrated to 2 × 10^4^ cells/mL in OGM medium supplemented with Differentiation SingleQuots (Lonza, USA). Next, cell were seeded on sterilized discs—test samples. Bottom of the well plate (TCPS) was used as a positive control. Cells were cultivated up to 21 days; cell viability/cytotoxicity tests were conducted at day 3 and 7, while cell mineralization was assessed at days: 7, 14 and 21.

#### Assays for viability and biomineralization

Cell morphology was evaluated using optical fluorescence microscope (Olympus, Japan). Cells were stained for 0.5 min with fluorochrome—acridine orange (AO), and then rinsed with phosphate buffered saline (PBS).

Cell viability was determined using ViaLight assay (Lonza, USA). The intensity of bioluminescence was related to the ATP concentration. Cytotoxicity of electrospun membranes was determined using ToxiLight assay (Lonza, USA) based on the quantitative determination of adenylate kinase (AK) released from damaged cells.

Mineralization in cell culture was assessed by OsteoImage test (Lonza, USA) based on fluorescent staining of hydroxyapatite deposited by cells. Cells were cultured using differentiating medium for 7, 14 and 21 days and the fluorescence of hydroxyapatite deposited by cells was measured at excitation wavelength λ = 492 nm and emitted wavelength λ = 520 nm.

The ALP activity was detected using 4-Methylumbelliferyl Phosphate (4-MUP) as a substrate. Cells were seeded on the biomaterials as described above. After 7 and 14 days in culture cells were washed with buffer and frozen and thawed three times to obtain cell lysate. The lysate was incubated with 4-MUP at room temperature for 1 h. Fluorescence of methylumbelliferone (4-MU) generated by ALP was measured at ex/em 360/440 nm and calculated into μm per μm of total protein content.

The intensity of bioluminescence and fluorescence of performed tests was measured using a PolarStar Omega microplate reader (BMG Labtech, Germany).

Statistical analysis was performed using Tukey’s test. Statistically significant differences were determined for *P* < 0.05.

## Results and discussion

### Morphological, chemical and mechanical characterization of electrospun scaffold

**Fig. 1:**
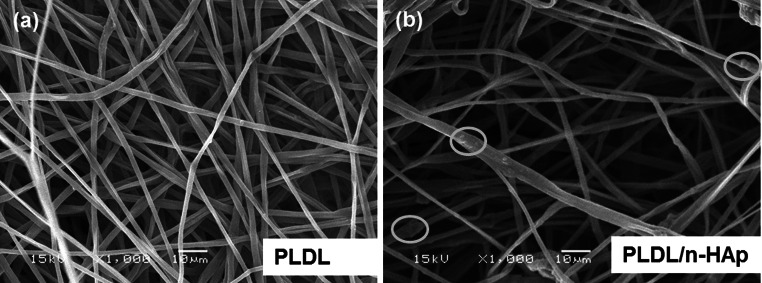
Microstructure of electrospun scaffolds: **a** PLDL; **b** PLDL/n-HAp

**Fig. 2:**
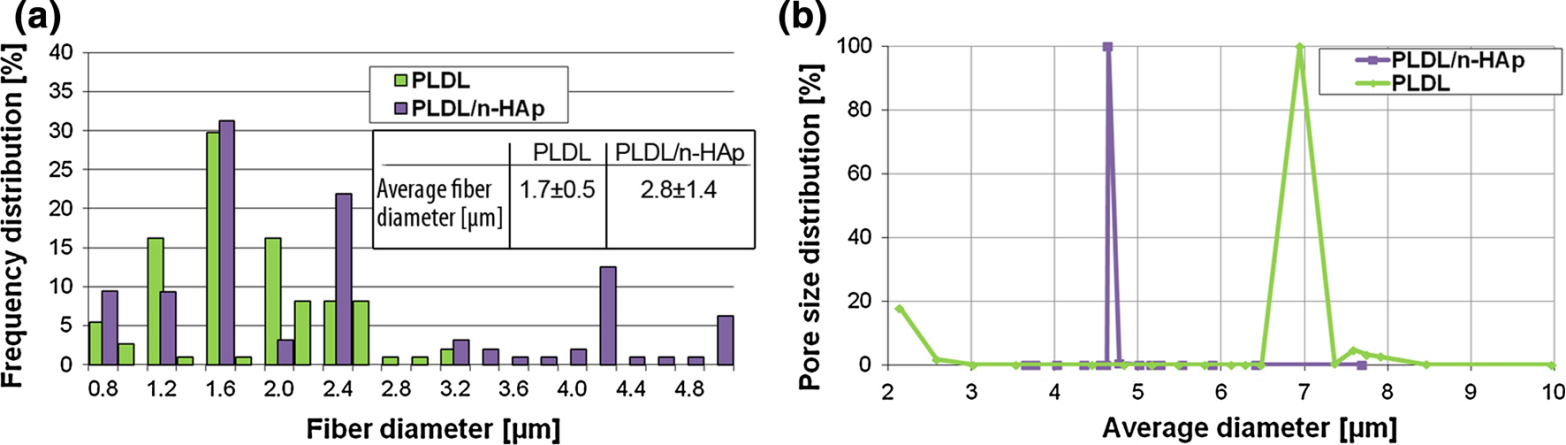
**a** Fiber’s diameter distribution and **b** Pore size distribution for PLDL and PLDL/n-HAp scaffolds

Electrospun PLDL and PLDL/n-HAp membranes are composed of smooth and uniform fibers with minimal bead formation (Fig. 1). The distribution of n-HAp particles in the PLDL matrix is nonhomogeneous and agglomerates of HAp particles were observed on the surface of PLDL/n-HAp fibers (Fig. 1b). n-HAp crystals at higher concentrations (at and above 20 wt %) have a tendency to form agglomerate (do not form homogenous colloidal suspension), thus subsequently are incorporated within the fiber structure during electrospinning. The fiber diameter of PLDL membranes is in the range from 0.40 μm to 3.2 μm (the average fiber diameter is 1.7 ± 0.5 μm), while PLDL/n-HAp fibers showed has diameter from 0.40 μm to 5.0 μm (the average diameter: 2.8 ± 1.4 μm) (Fig. 2a). The membranes are porous and pores are interconnected. The main pore fraction for PLDL membrane is in the range of 6.5–7.5 μm, and the PLDL/n-HAp scaffold showed narrow distribution of pore size centered at 4.8 μm (Fig. 2b). The decrease in porosity of the composite PLDL/n-HAp membranes can be attributed to the effective charge dissipation by HAp particles, preventing inter-fiber repulsion [15].

**Fig. 3:**
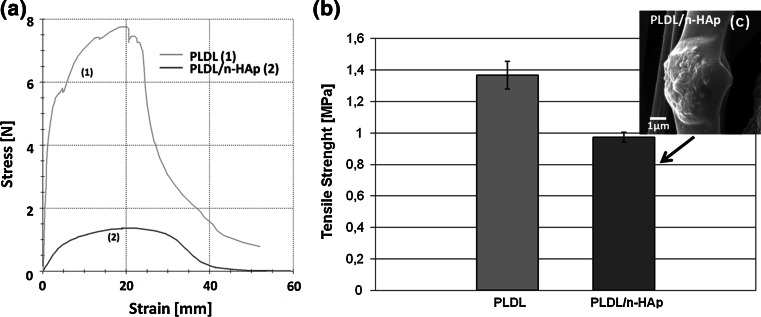
**a** Stress–strain curves obtained from tensile tests performed on electrospun PLDL and PLDL/n-HAp nonwovens; **b** tensile strength of electrospun nonwovens; **c** SEM micrograph of HAp agglomerates on PLDL/n-HAp fibers’ surface

Thomas et al. [28] reported that small concentrations of n-HAp result in a reinforcing effect on the polymer matrix. However at higher n-HAp concentrations no further improvement of mechanical properties were observed. In our case HAp agglomerates resulted in 30 % drop of the mechanical properties of PLDL/n-HAp membranes when compared with pure PLDL membranes (Fig. 3).

**Fig. 4:**
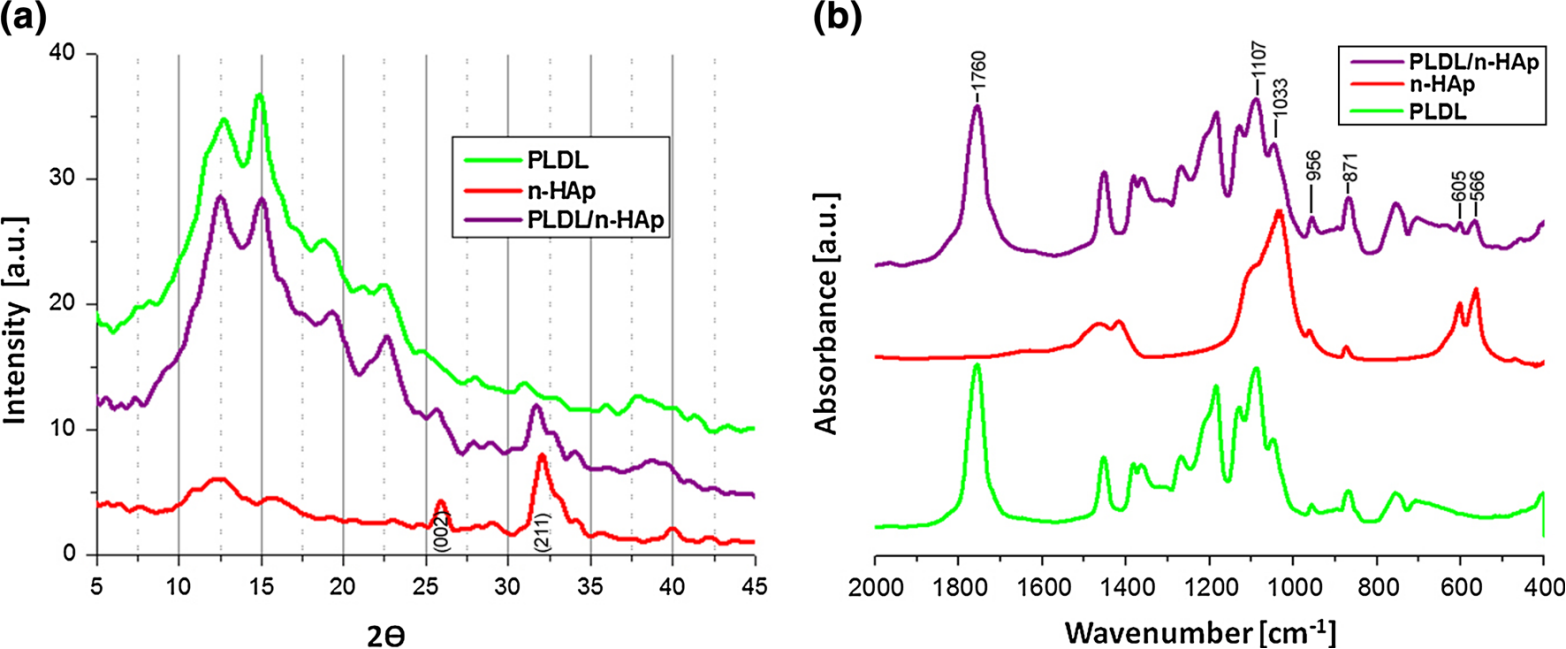
The comparison of: **a** WAXD patterns and **b** FTIR spectra of PLDL, PLDL/n-HAp and n-HAp samples

WAXD patterns of pure PLDL, composite PLDL/n-HAp and n-HAp powder are shown in Fig. 4a. The strongest intensity peak at 31.8° and the peak at 25.9° on the n-HAp powder WAXD pattern, corresponds respectively to the (211) and (002) diffraction peaks of the hydroxyapatite crystalline structure [29]. It should be notice that the both diffraction peaks also occur on the WAXD pattern of PLDL/n-HAp composite. This is a strong evidence of HAp presence in the composite membrane structure.

The FTIR spectra of n-HAp, PLDL and PLDL/n-HAp composite membranes are presented in Fig. 4b. The C=O absorbance band at 1,760 cm^−1^ in the poly(l-d-lactide) chains has been used as a typical characteristic band, which can be observed in both PLDL and PLDL/n-HAp samples. The characteristic bands corresponding to the deformation vibrations of PO_4_^3−^ (566, 605 cm^−1^), which do not appear in the case of pure PLDL, can be identified in PLDL/n-HAp samples. Also higher intensity of several absorption bands from PLDL at 871, 956, 1033, 1107 cm^−1^ corresponding to the vibrations bands of PO_4_^3−^ and CO_3_^2−^ group was observed. The FTIR and WAXD results confirmed that n-HAp particles were successfully incorporated into the electrospun composite membrane.

### Biomineralization

Mineralization was assessed in vitro using two concurrent methods: (1) simulated body fluid (SBF) [30] and (2) cell based assays.

#### Mineralization in SBF

**Fig. 5:**
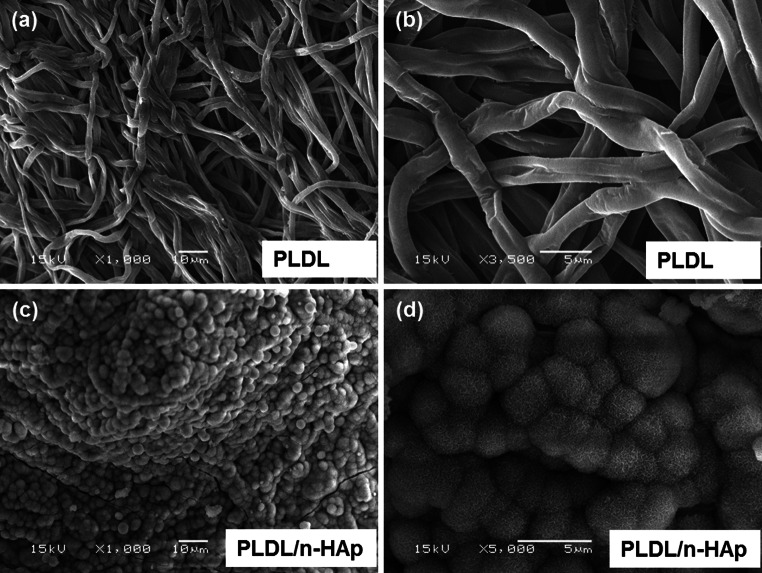
SEM micrographs of **a–b** PLDL and **c–d** PLDL/n-HAp scaffold after 7 days immersion in SBF solution

**Fig. 6:**
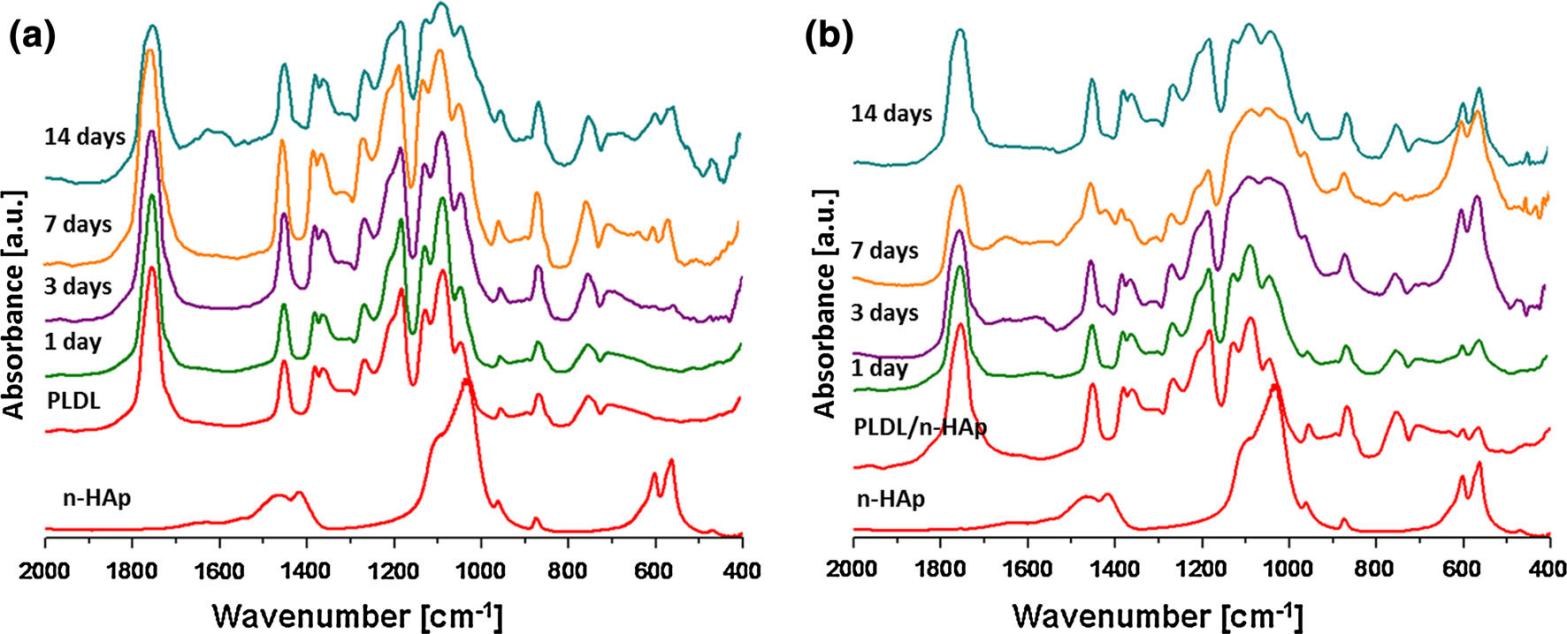
FTIR spectra of: **a** pure PLDL (as reference) and **b** PLDL/n-HAp scaffolds after different time of immersion in SBF solution

**Fig. 7:**
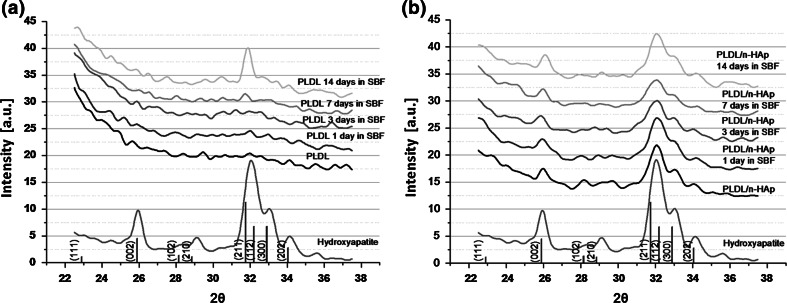
WAXD patterns of **a** pure PLDL (as reference) and **b** PLDL/n-HAp scaffolds after different time of immersion in SBF solution

The mineralization of PLDL and PLDL/n-HAp scaffold was investigated in 1.5 × SBF. n-HAp particles present on the surface of PLDL/n-HAp membranes acts as nucleation sites for mineralization in SBF (Fig. 5). It was observed that after 7 days of soaking a layer composed of spherical aggregates (characteristic for apatite precipitated from SBF) was formed on composite membrane surface (Fig. 5c–d). For PLDL samples no apatite deposits were observed (Fig. 5a–b). The presence of crystalline apatite on the surface of PLDL/n-HAp membrane was confirmed with FTIR at day 3. While for pure PLDL samples only very week bands associated with carbonated calcium phosphate were detected after 7 days (Fig. 6). The WAXD diffraction spectra were collected for both PLDL and PLDL/n-HAp samples after different time-point of immersion in SBF (Fig. 7a). After 7 days in SBF, PLDL samples showed negligible amounts of poorly crystalline apatite (211) diffraction peak at 31.8°. After 14 days this peak increased which suggested the formation of the uniform apatite layer. While for PLDL/n-HAp membrane, the sequence of characteristic hydroxyapatite diffraction peaks (002), (211), (300), (202) was detected on WAXD pattern at 25.9°, 31.8°, 32.9° and 34.0° [29] which confirmed the formation of crystalline apatite layer that indicates that the membranes are capable of inducing mineralization in bodily conditions.

#### Viability and mineralization in cell culture

**Fig. 8:**
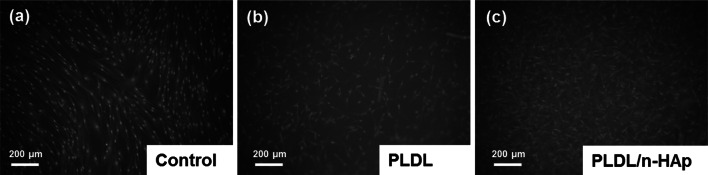
Morphology of NHOst cell cultured for 7 days on the TCPS control surface and on the surface of PLDL and PLDL/n-HAp electrospun materials. Scale bar is 200 μm

**Fig. 9:**
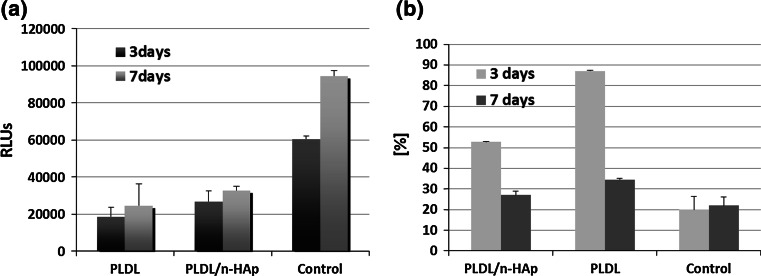
**a** NHOst cell viability measured by the Vialight test on the 3rd and 7th day of the culture on the TCPS and the surfaces of electrospun PLDL and PLDL/n-HAp nonwovens. Statistically significant differences between the control group and the materials examined according to Tukey’s *t* test for *P* < 0.05. The figure shows cell viability in relative luminescence units (RLUs); **b** percentage value of cytotoxicity in respect to viability of cells cultured on studied biomaterials

The microscopic observation showed that NHOst cells attached well both to PLDL and PLDL/n-HAp membranes however larger number of cell was present on composite material. The cells are well spread with long and slender extensions (Fig. 8). Due to the porosity of the membrane, the network of cells connected by cytoplasmic extensions has a three-dimensional structure. Such kind of cell behaviour confirms that obtained materials create appropriate environment for bone cell growth and tissue formation. The results of viability of the cells seeded on electrospun membranes didn’t show any significant difference between materials (Fig. 9a). Moreover, a lack of major differences in cells viability between 3 and 7 days series both for PLDL and for PLDL/n-HAp is probably an effect of stabilization of culture caused by cells differentiation. Probably the fibrous microstructure of electrospun membranes affects cell viability, which is much lower compared to the control flat surface (TCPS) extremely favorable for adhesion and intense proliferation of cells. Cytotoxic influence of membranes was calculated as a percentage value of cytotoxicity in respect to viability of cells cultured on studied biomaterials (Fig. 9b). The values of cytotoxic test were lower for PLDL/n-HAp membrane compared with pure PLDL membrane. That means the presence of n-HAp in the composite membranes positively influenced cell viability.

**Fig. 10:**
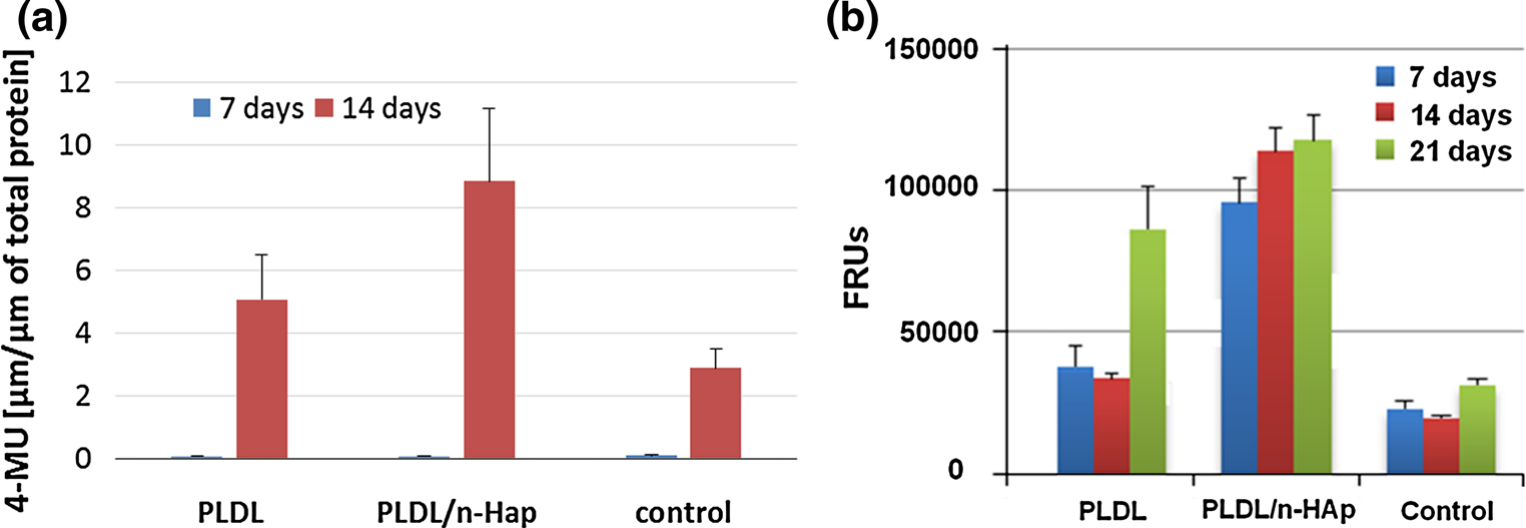
**a** ALP activity; **b** Mineralization progress measured by the concentration of hydroxyapatites on the 7th, 14th and 21st day of NHOst cell culture on the TCPS and on the surface of the nonwoven discs

The alkaline phosphatase activity (ALP) has a crucial role in the cell differentiation and initiation of the mineralization process [31]. High ALP activity was observed for both membranes, especially for PLDL/n-HAp (Fig. 10a). These data demonstrated that the cell differentiation is upregulated by the presence of n-HAp and by microstructure of the materials.

In order to evaluate the effect of n-HAp content on the mineralization process in the cell culture, OsteoImage (OI) test was performed. The rate of mineralization assessed by OI was the highest in the case of n-HAp modified membrane (Fig. 10b). In the case of PLDL membrane, the effect of material influence on mineralization process is significantly pronounced after 21 days. Calcium deposits obtained during cell culture suggested that composite membranes support in vitro osteoblastic differentiation.

## Conclusions

Novel PLDL/n-HAp composite membranes for bone tissue engineering were successfully produced by electrospinning. The incorporation of the n-HAp into the structure increased significantly the mineralization of the membrane in in vitro conditions. It has been demonstrated that after a 3 day incubation in SBF a continuous compact apatite layer was formed on the surface of the composite membrane. The membrane microstructure and chemical composition were found to have positive effect on cells attachment, proliferation and morphology. In vitro experiments using NHOst cells showed that the addition of n-HAp provided chemical cues that was a key factor that regulated osteoblastic differentiation. In summary, preliminary studies have shown that PLDL/n-HAp electrospun scaffold can direct HAp mineralization both in SBF and in cell culture. This study formed a strong foundation to design osteogenic scaffolds for bone tissue regeneration.
